# Promoting Public Engagement during the COVID-19 Crisis: How Effective Is the Wuhan Local Government’s Information Release?

**DOI:** 10.3390/ijerph18010118

**Published:** 2020-12-26

**Authors:** Yi Yang, Wen Deng, Yi Zhang, Zijun Mao

**Affiliations:** College of Public Administration, Huazhong University of Science and Technology, Wuhan 430074, China; yiyangcpa@hust.edu.cn (Y.Y.); yizhanghn@sina.com (Y.Z.); maozijun@hust.edu.cn (Z.M.)

**Keywords:** social media, COVID-19, public engagement, crisis communication

## Abstract

During times of public crises (such as COVID-19), governments must act swiftly to release crisis information effectively and efficiently to the public. This paper provides a general overview of the way that the Wuhan local government use Weibo as a channel to engage with their citizens during the COVID-19 pandemic. Based on the media richness, dialogic loop, and a series of theoretically relevant factors, such as content type, text length, and information source, we try to examine how citizen engage with their local government. By analyzing the data mining samples from Wuhan Release, the official Sina Weibo account of Wuhan’s local government, results show that, despite the unstable situation COVID-19 over the crisis, there exist three stages of a crisis on the whole. Combining the behavior of the government and the public, duration from 31 December 2019 to 19 January 2020 could be seen as the development period, then the outbreak period (30 January 2020 to 28 February 2020), and a grace period (29 February 2020 to19 April 2020). Public attention to different types of information changes over time, but curbing rumors has always been a priority. Media richness features partially influent citizen engagement. Text length is significantly positively associated with citizen engagement through government social media. However, posts containing information sources have a negative impact on citizen engagement.

## 1. Introduction

In the past decade, social media (SM) has been playing an increasingly important role in modern society. For the government, these innovative applications based on this platform can release a large amount of information for a wide audience in real-time at a relatively low cost [1], providing opportunities for dialogue between agencies and citizens [2,3]. Social media can not only enhance the transparency of government departments, but also improve public service delivery and public policymaking [2]. Eltantawy and Wiest [4] point out that providing support to the public and the public sector is the real potential source of social media. Therefore, social media can be seen as a communication tool to promote public engagement through effective feedback, coordination, and discussion.

Coronavirus disease (COVID-19) is a new infectious disease transmitted mainly through respiratory droplets and contact, which poses a great threat and challenge to public health. China’s first case of COVID-19 occurred in Wuhan, Hubei province. The Novel Coronavirus outbreak was listed as a public health emergency of international concern by the World Health Organization (WHO) on 30 January 2020 [5]. In the process, information and discussions about COVID-19 spread rapidly across the Internet, especially on SM.

At present, social media has become an important channel to promote risk communication during the epidemic crisis [6,7]. Increasing numbers of public health departments and individuals tend to use social media platforms to communicate and share information. In the previous literature, many studies have applied social media data to infectious disease studies in the context of public health as a measure of public engagement. For example, H7N9 [8,9,10], Ebola virus [6,11,12,13,14], Zika virus [7,15,16], etc.

As a main social media platform in China, Sina Weibo has become increasingly important for the public to spread epidemic diseases since the outbreak of the Novel Coronavirus. It is an interesting and urgent issue to study the extent to which the public is concerned about the COVID-19 epidemic on Weibo, to promote communication between the government and the public through risk communication and community participation, and to eliminate public confusion and misunderstanding.

However, there are a limited number of studies on Weibo in the public sector or at local government, most of them focus on the analysis of SM (mainly Twitter) for electoral campaigns [17,18,19,20,21]. Therefore, we want to analyze citizen engagement on the social media platform, not only for political purposes but also for social mobilization. The open-source of platform and computing techniques make it possible to research this phenomenon of engagement, to evaluate public opinion. Several scholars have pointed out the importance of focusing on what local entities do on SM to assess the real impact on government-to-citizen (G2C) relationships [2,22].

The main purpose of our study was to analyze how the Wuhan local government use Weibo to interact with their citizens during the COVID-19 epidemic. As the only official Weibo account of the Wuhan government, Wuhan Release has 3.78 million followers (as of September 2020), and has a large number of posts every day. We focused on Weibo because it is one of the most popular SM platforms in China, which is the equivalent of Twitter. As of the fourth quarter of 2018, the number of monthly active users had reached 462 million, and approximately 200 million people are using Sina Weibo every day [23]. It offers opportunities for organizations to contact different types of individuals and broadcast information [24]. In the past, Weibo experienced a steady growth, while recently the number of active users is rather stable, and not the microblogging site has created a great community and shows strong loyalty [25].

This study attempts to indicate which factors in Wuhan Release’s posts influenced citizen engagement on Weibo, and consider the following: content type, text length, information source, media richness, the dialogic loop of the text. In the following research, we tried to answer these two questions:

**RQ1:**  *How different information factors of Weibo posts (content type, text length, information source and media richness) from Wuhan local government information release influence citizen engagement during the COVID-19 pandemic?*

**RQ2:**  *To what extent does the dialogic loop of the Wuhan local government information release influence citizen engagement during the COVID-19 pandemic?*

To our best knowledge, this is the first study on COVID-19 to provide a general overview of Weibo usage by Wuhan local governments, offering insights into the correlations related to indicates such as the published posts, contents type, and the citizens’ engagement. Due to the lack of empirical studies, this study would help local governments to integrate academic research into the local communication strategy, we believe that our paper might have implications for both research and practice as it provides an overview of how the Wuhan local governments use Weibo, and what factors influence citizens’ responsiveness.

To boost engagement, understanding what content types attractive is important [1]. Some particular content types might be more engaging than others [26]. Hence, identifying those types together will be useful in maintaining a fluid conversation with citizens during a crisis [27]. Once the strategy and factors are identified, guidelines should be established, which would be beneficial to both sides, government, and citizens.

This study combines both quantitative and qualitative data. This mixed-method has been proven to be effective in previous studies [28]. The quantitative component focuses on the statistical analysis of metrics related to follower behavior (reposts, replies, likes) to understand the level of engagement. The qualitative analysis is drawled on grounded theory, coded the text to identify the content of posts and concluded into various topic categories [29]. By identifying the factors related to Weibo citizen participation, we analyzed whether different content and media characteristics influence the level of engagement, as well as the significant relationship.

This paper is divided into five parts. The second part is a review of previous research. The third part describes the methods of our research. The fourth part presents the results of the research on citizen engagement in the government of Wuhan. The last part justifies the main findings of this paper, discusses the limitations of the study, and looks forward to the future research direction.

## 2. Literature Review

### 2.1. Government Information Release and Crisis Lifecycle

SM has emerged as an important medium for governments and citizens to capture and explain crises, and take action accordingly [30]. On the one hand, SM platforms allow citizens to search and share reliable information. On the other hand, SM can enhance citizens’ abilities in understanding present situations and solving problems collaboratively due to its powerful capacity for inter-connectivity. Therefore, a two-way, responsive, cyclical communication process is obtained. The government can eliminate the middlemen in dialogue communication (such as journalists) [31]. Currently, public agencies are increasingly integrating SM into local administration communication strategies and interacting directly with citizens [32]. Especially when a crisis happened, individuals who access news would like to follow the principle of proximity. A national survey indicated that during the COVID-19, the Wuhan citizens, as the most suffered group, trend to pay close attention to the local news release [33]. This not only bring challenges to the local government, but also gives them opportunities to better handle the crisis response.

SM could capture the change of mood, values and attitudes of the public, which is particularly important for managers to understand the importance of social media platform [34]. During emergency management, with the help of SM, teasing out the timing of the crisis and understanding that kind of information is useful to the public would be helpful for the crisis management job. In a public crisis, the identification of what kind of information the public need is an essential aspect of the emergency response. It will also be important to define the different phases based on the behavior of the public and the characteristics of the event. According to Fink [35], crisis management focuses on a comprehensive process, which also is described as the theory of crisis lifecycle. Crisis moves through several stages with disparate characteristics and requirements for governments and first responders. There are various theories of the communication and crisis development of, for example, the three-stage model [36], the four-stage lifecycle [35], or the five stages of a crisis lifecycle [37]. Coombs provides an overview of his own three-stage crisis development model: pre-crisis (detection, prevention, and preparation), crisis (recognition and containment), and post-crisis. Some scholars differentiated four stages in a crisis lifecycle: two pre-crisis phases (mitigation and preparedness) and two post-crisis phases (response and recovery) [38].

In this research, we refer to the three-stage model due to its simplicity and the specific-stage features. The pre-crisis occurs when an impending or potential crisis is detected, the actors start to prepare and prevent the event. The second stage is the extreme of the crisis, crisis response occurs, and the authorities implement crisis reduction plans and seek to control the damage. The third and final phase emphasizes recovery, inventory, and learning. The purpose to divide crisis into several stages is focusing on how the authority and the public behave on the social media platform during the different stages of COVID-19 might help to understand the crisis itself. In the first stage, people are in an incubation state, in the second stage the greatest damage is inflicted and the authorities take more extreme mitigation and containment responses. The final stage starts the progress of recovery and returns to the normal.

The public needs of information shift constantly in response to the spread of the epidemic. For example, in the early stages of SARS, because of the contagious and insidious nature of the virus, the public wanted to know the truth about the outbreak and preventive measures related to their own safety. During the outbreak period, the public was concerned about the latest data on the outbreak and the government’s response. While at the end, the public turned to the attribution of responsibility and the development of vaccine drugs [39]. During the Zika epidemic, the public mainly focus on the access to the truth, effective government decision-making, ways to participate, resource allocation and maintaining a normal life [40]. Therefore, we come up with the first hypothesis:

**Hypothesis 1.**  *The type of content that the public is most interested in has changed over time.*

### 2.2. Dialogic Loop and Citizen Engagement on Social Media

If a government aims to achieve more citizen engagement, it must go beyond more publication of information [41]. Similarly, Zavattaro and Sementelli [42] claim that one-way communication cannot boost interaction, nor lead to an engaged society. Nevertheless, Mergel [41] has shown that public administration is still inclined towards a unidirectional communication model on SM.

Dialogue is regarded as “negotiated exchange of ideas and opinions” [43]. The dialogue can be incorporated into daily public relations practices, which could lead to positive outcomes, such as trust, mutuality, and empathy [44]. According to the theory, dialogic loop is regarded as a key dialogic principle for a positive two-way dialogic relationship [43], which refers that the content released by organization could stimulate public dialogue, and the organization could raise questions to promote engagement, adding providing the dialogue channels and responding to public feedback timely [27]. Dialogic loop could be embodied by many terms, e.g., with the use of hashtags and the @function [27,45], it is visible in the commenting and responding functions of the social media [46].

Few studies have indicated how organizations maintain dialogic relationships and promote public engagement through social media such as Facebook [47] and Twitter [48]. There is little empirical evidence of the actual impact achieved by the dialogic loop on levels of engagement in the Chinese context. Citizen engagement has been defined as the individual or collective behavior aimed at resolving social problems in the community [49,50]. The essence of it is in the interaction between citizens and the government [51]. By using this term, academics or professionals might refer to stakeholder involvement, co-creation, political participation, citizen engagement, participatory democracy, or activism [50,52,53,54,55]. Given the important role it plays in the social system, citizen engagement is a complex phenomenon that deserves further studies [56]. SM empowers the capacity of engaging people and organizations [42]. It has enabled wide engagement due to its ubiquitous and real-time access to information. People can share knowledge and situational awareness clearly and effectively through the government release on online platforms (uploading photos and records), thus promoting collaboration between citizens and the government.

Nowadays, the adoption of SM by governments has attracted the attention of academics and much research on this subject has been conducted [57,58,59,60] following diverse methodologies [32]. Several authors [61,62,63] have pointed out the importance of SM as a key tool to boost communication and dialogue with citizens. According to several authors [50,53,60,64], the use of SM positively influents citizens’ engagement. Nevertheless, Bonsón et al. [61] argue that only SM is not enough, and local governments must learn online communication strategy (like dialogic loop) and encourage two-way communication between citizens. Similarly, Skoric et al. [65] note that although the relationship between the use of SM and participation is crucial, citizen engagement is more complex than thought. Therefore, we come up with the second hypothesis:

**Hypothesis 2.**  *Dialogic loop (the use of hashtag and @function) would generate a higher engagement rate.*

### 2.3. Media Richness and Information Influencing Factors

Daft and Lenge [66] firstly come up with media richness theory, which gradually became popular with the emerge of electronic media. Media Richness theory regards communication channels as possessing many characteristics to carry information. The rich information is more able to reduce equivocality than lean information [66]. As a theory which refers to the relative ability of a communication channel to deliver messages [67], it includes four dimensions: (a) immediate feedback, (b) multiple cues, (c) language variety, and (d) personal focus [68,69].

Media richness theory has been used in multiple fields. In computer science fields, integrated media richness theory is used combined with flow theory, and the technology acceptance model to investigate how they can explain the acceptance of e-learning technology [70]. It is also an important factor influencing the customer experience in e-commerce [71]. This theory represents the structure of richness (e.g., multiple cues and responsive feedback) to determine the level of richness. Moreover, it also has been used to indicate the effect of different types of communication media (see, e.g., [72,73]).

However, on social media platforms, media richness theory research generally focuses on communicators’ choices of channels (e.g., a face-to-face meeting vs. a teleconference), few of them study whether rich media influent the effect of information transfer [66,74,75]. For example, visual images are more powerful tools for communicating messages regarding all aspects of organizations [44]. In particular, if a website uses multimedia functions and frames, it would have a higher level of media richness than text alone.

The multi-perspective medium could affect the effectiveness of the information disclosure. In other words, media richness theory assumes that media vary in degree of “richness”, or the “ability of information to change understanding within a time interval” ([66], p. 561). Thus, “richer” media are characterized by the use of responsive feedback, multiple cues, appropriate use of language, and a “tailored frame of reference” [76].

As discussed above, SM allows diverse multimedia content to be published, including images, videos, or hyperlinks. The importance of media for conveying information has been highlighted [77,78]. They argue that plain text is no longer the best type of medium to articulate information, and that multimedia is the most effective way to enhance the potency of a given message. Media analyses of Facebook also conclude that the use of pictures encourages citizen interaction, with more likes and comments than text-only publications [1,59]. Similarly, a media analysis study on Twitter [79] also shows that messages with a high level of richness, such as images and photos, tend to have a greater response from citizens. Therefore, we come up with the following hypothesis:

**Hypothesis 3.**  *Higher media richness (picture, video, web-links) would generate a higher engagement rate.*

In addition, we also assume that other information influencing factors potentially affect public engagement, which are as follows:

Text length: While some scholars found the relationship between text length and engagement, some research indicates that longer text could get more attention [80]. For example, an organization’s lengthy tweet could significantly reduce the negative sentiment of customers’ tweets [81]. It could be explained that longer sentence requires more time and resources, so it might positively influent users’ engagement and develop a strong online community [82]. Li et al. [83] found that, during the COVID-19 pandemic, the content length of most types of situational information has a positive correlation with the propagation scale. Another researcher assumed that a longer length content may include enough detail and have been recognized to positively affect the feedback [84]. Therefore, we come up with the following hypothesis:

**Hypothesis 4.**  *Longer posts would generate more engagement than shorter posts.*

Information source: In addition to releasing original information, the official account could also be operated as a news aggregator. Because of multiple source layers in online information transmission, the official account frequently cites some important information from other sources. Compared to the original source (the account that is actually responsible for creation of the core content), there are another type of posts with visible source, which can be defined as “the source seen by the receiver to be delivering the message or content” [85]. Until now, it is unclear that whether a post is original or not would influence the public engagement. Some similar research found that people are more likely to comply with the direct message from official source [86]. When facing a crisis or disaster, local departments were more important sources than routine times, and people regarded local government sources as references [87,88]. Therefore, we come up with the last hypothesis:

**Hypothesis 5.**  *The original post will lead to more engagement than posts from the other source.*

## 3. Method

### 3.1. Research Case: COVID-19 Crisis in Wuhan

In December 2019, hospitals in Wuhan, Hubei province, China, identified several unknown pneumonia cases, which were later confirmed to be caused by a new type of coronavirus. Then the World Health Organization (WHO) renamed this disease, COVID-19 on 11 February 2020. This study took this public health crisis as a case due to its global pandemic nature and grave threat to human life and health. Wuhan, as the early city to be affected, has gradually recovered from the crisis. The experience from Wuhan would be helpful and enlightening for the other regions which still suffer the COVID-19 heavily. After the outbreak of COVID-19, official Weibo accounts were one of the most important sources of crisis-related information [89]. There were few studies focused on the information release and crisis response during the COVID-19 [27,90], but there is a lack the research about the communication behavior of the local government and the citizens’ reaction during the COVID-19. This study focuses on the official Weibo account Wuhan Release of Wuhan’s local government. There are three reasons for this: first, Weibo is one of the most popular SM platforms in China and has the first series of government accounts with a large number of followers (more than 3.78 million). Second, people prefer official news sources with strong authority and credibility during a crisis, and the Wuhan citizens were more likely to choose local media as their main source [33], thus Wuhan Release played an irreplaceable role in crisis communication to Wuhan citizens. Third, there exists the possibility that the government may regulate the content on social media by deleting or screening negative, sensitive, and extreme comments [91], which could influence the validity and reliability of the data from social media. We choose an official outlet is that it could relatively relieve the influence from the Internet censorship, that’s because the official media sifted through the information at the time of its release, and the research data would not be heavily influenced by the sensitivity of the topics discussed and the division of opinion [92].

### 3.2. Sampling and Data Collection

Data in this study were collected from the ‘Wuhan Release’ Sina Weibo account of the Wuhan government. It has firstly reported viral pneumonia of unknown causes on 31 December 2019. At this time, the online reaction of the public may be greatly intensified as they were faced with this severe condition suddenly. After referencing to other studies [27,93], with the help of python toolkit, we crawled all related posts during the period of 31 December to 19 April 2020, from the day that the earliest cases in Wuhan were reported, to the day that the number of confirmed, suspected cases were cleared. The data includes the publisher, title, text length, information source, number of reposts, comments, likes, and whether @somebody/#hashtag# is contained, which was acquired and stored according to the time. At the same time, the URLs of the pictures or videos uploaded were also captured, and we also determined the text type (i.e., whether or not the account was actively using pictures or videos). A total of 3596 posts were pertinent to COVID-19 after a manual check.

Most studies have evaluated engagement using quantitative indicators of social media platforms, like the number of repost, comments, and likes [94,95,96] (Bonson and Ratkai, 2013; Agostino and Arnaboldi, 2016; del Mar Galvez Rodriguez et al., 2019). These three indicators respectively reflect the engagement behavior of the users. For instance, the share (repost) activity in crisis could be seen as an informal recommendation system for the content [97,98]. Then we calculated the number of comments below each post, the public who reply want to express their concerns, respond to the authorities, and reveal subjective feelings [99]. Finally, we are concerned about the thump up behavior (likes), which could be regarded as a supportive behavior, as one of the public engagement factors. These data were the factors related to the citizen engagement (repost, comment, likes) that were studied through a statistical analysis with these factors related to the content type, dialogic loop, media richness, and text length et al., to understand the influence between citizen engagement and the government information release.

### 3.3. Content Analysis

Content analysis is defined as a multi-purpose research method to study a wide range of issues by systematically, complex, and objectively identifying characteristics of large sample data [100]. Followed the grounded theory approach, in spite of collecting data, we also need to generate concepts and topics from the ground up [29,101]. During this analysis, two trained coders conducted the coding work. The first step was defining the coding norms, and constructing categories as content type. After examining inter-coder reliability, the two coders started to analyzing data. The reporting results are shown in Table 1.

## 4. Results

### 4.1. Descriptive Statistics of the Content Type and Crisis Lifecycle

**Figure 1:**
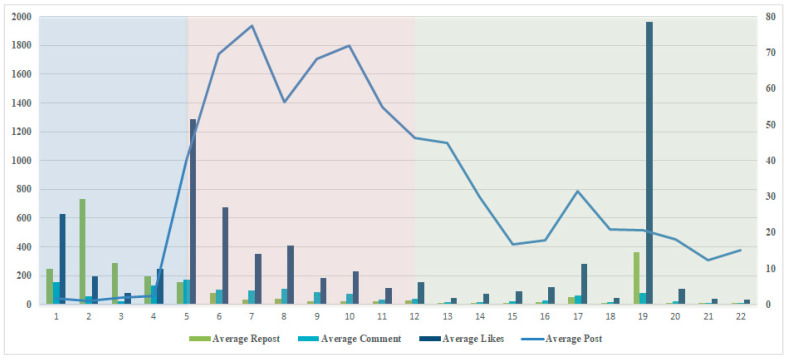
Average count of each days’ post/repost/comment/likes during 22 groups.

To analyze the long-term data more intuitively, we divided the duration from 31 November 2019 to 19 April 2020 into 22 groups (Table 2), each group contained five days. After this step, we calculated the average count of each day’s post of the Wuhan Release of each group. Then, we obtained the count average count of the repost, comments, and likes of each post of each group, and to avoid the influence of abnormal value, we remove the extremum of the repost, comment, and likes. Even so, there were some extreme values in Figure 1, and we will take this into account in our analysis.

Through Figure 1, we could roughly divide the crisis into three stages. Considering the characteristic of the epidemic crisis and the physical truth of the COVID-19, there are some differences with Coomb’s three-stage crisis development model. We describe the pre-crisis as the development period, the crisis as the outbreak period, and the post-crisis as a grace period.

Combining the behavior of the government and the public, we distributed the first 4 groups (31 December 2019 to 19 January 2020) as the development period, between group 5 to group 12 is the outbreak period (20 January 2020 to 28 February 2020). And the period from 29 February 2020 to 19 April 2020, the last 10 groups was regarded as a grace period. At the first stage, the public was aware of this new crisis and showed considerable attention, they tend to repost to tell others some news of the new unknown virus. However, when no more information came out, public attention was not continuable and phased down quickly. This was later seen as a failure of crisis management in the early stage. With the outbreak period, both the government and the public paid much more attention than before, people would like to talk with others through online comments to gather more information, and relieve their emotion or support the government action through thump-up. As the crisis became under control and the situation was improved, although there were some small range fluctuations, the crisis came into a grace period as a whole. However, people in this period were vigilant about the crisis, any sensitive information could trigger a large-scale spread. For example, in group 19, there was a rumor about new cases in Wuhan, people panicked again, and when the government quickly clarified the fake news, the effect was stopped immediately.

The most popular content types among the Wuhan Release in our sample are shown in Table 3. The results show that the local government tends to use their Weibo account to thank the worker (*n* = 823, 22.89%) and report news (*n* = 795, 22.11%). Except for these two types, there also includes government measures (*n* = 536, 14.91%), scientific guidance (*n* = 446, 12.40%), epidemic data release (*n* = 396, 11.01%). Other categories which were less than 10% include: notice release (7.73%), encouragement (6.15%), and dispel rumors (2.81%).

Table 3 shows that the most frequently used media type is website links (35.68%). After that, the statistical results show that Wuhan local governments tend to use visual tools, such as photos (16.04%) and videos (16.04%). Moreover, 15.98% (*n* = 205) posts contained forwarding and 10% (*n* = 305) of @posts from other users communicated with the public. Finally, the total amount of traditional plain text posts is only 8.63% (*n* = 50), indicating that this is different from Bonsón et al.’s (2019) research results. That is to say, in the context of the COVID-19 crisis, Wuhan local government attaches great importance to promoting citizen participation through multiple media forms, rather than plain text.

### 4.2. Median Statistics of Content Type

**Figure 2:**
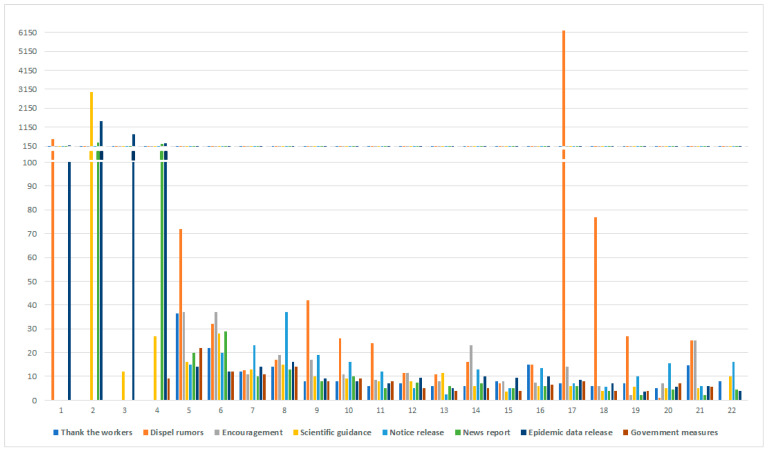
Median Repost count for 8 categories of content.

**Figure 3:**
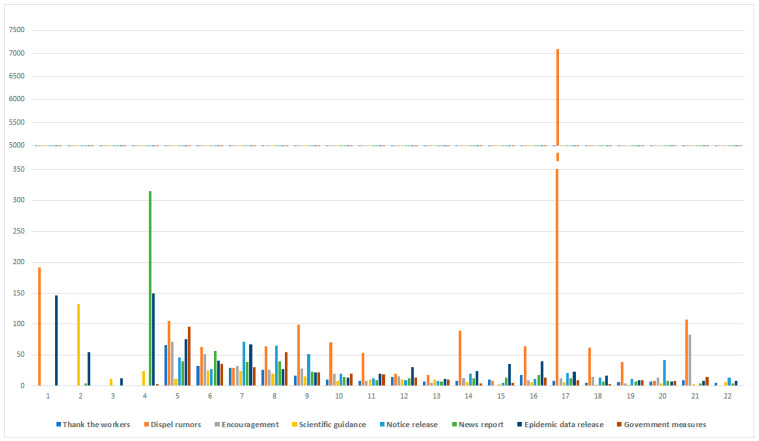
Median Reply count for 8 categories of content.

**Figure 4:**
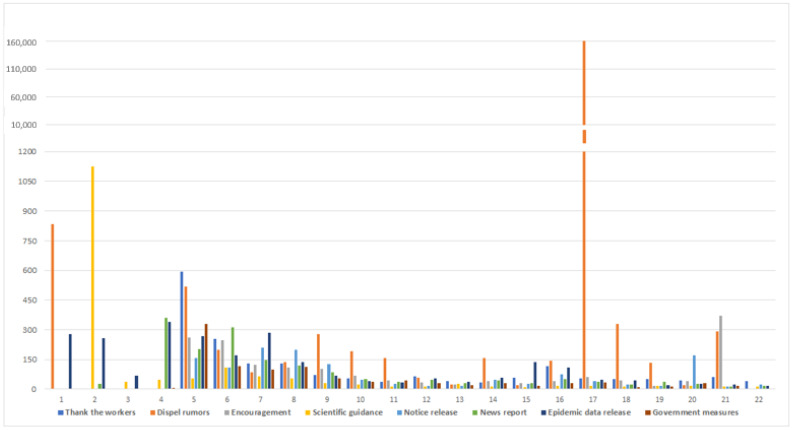
Median Likes count for 8 categories of content.

To determine whether different categories of content influent the public engagement during the crisis, we calculated median repost, reply, and likes across each group. These figures below (Figure 2, Figure 3 and Figure 4) show that public engagement is different across different time-line and categories. Whether in repost, reply, and likes, dispel rumors garners much more interaction than other categories during the period. Because the spread of rumors would damage the crisis prevention and control effects, and mislead the public, the authorities and the public pay much attention to dispel rumors. At the early stage of the crisis, on account of the unknowability of the virus, people tend to engage more in posts about scientific guidance, epidemic data release, and news report, to protect themselves effectively and repost this kind of message to let more people be aware of it. Thank the workers and encouragement content elicit repost and likes from the public in the middle and the later period. The funding could be explained that the crisis was toughly under control, people need to release their emotions and develop gratitude spontaneously. Research shows that these types of government measures generate considerable engagement during the middle stage, including repost, reply, and likes. Except the dispel rumors and encouragement, other categories cannot trigger too many citizens’ interest to reply and likes during the last period of the crisis.

Back to Hypothesis 1, we found that with time-varying, the public most interested content type has indeed changed. At the early stage of the crisis, most people were concerned about the introduction of the virus and the method to prevent this new epidemic disease. During the development of the crisis, citizens paid much attention to the government’s employment and relevant notice. At the end of the crisis, the release of emotion and gratitude became mainstream. Control rumors were the mainstream during the whole crisis.

### 4.3. Multivariate Statistics

Table 4 shows the results of our correlation model predicting Wuhan’s local government and citizen engagement during the COVID-19 crisis. Hypothesis 2 focused on whether the level of citizen engagement depends on the dialogic loop. The correlation model showed posts related to the COVID-19 crisis that contain “@other accounts” positively improve citizens’ reposts and likes, which indicated more interactions between different accounts. However, at the same time, none of the indicators of hashtag were significant. In summary, the dialogic loop factors are partially associated with citizen engagement.

Hypothesis 3 proposed that whether Weibo posts presented with media richness features of Wuhan Release tended to lead to more citizen engagement. Among them, the increase of pictures brought a significant increase in comments, because the pictures could deliver more information than text could convey, people were willing to express when they saw one or more pictures. However, the existence of video led to a decrease in comments. The difference between picture and video could explain this phenomenon, unlike the picture’s simply and visualized, learning the message of a video could waste more time, some people were unlikely to open the video or watch the full video, so they also showed no interest to discuss the video. The existence of links led to a significant increase in of likes, reposts, and comments, which means people tend to engage with information with reliable evidence. On the other hand, only important information needed to be explained further by linking. In consideration of the relationship between media videos and citizen engagement had a negative correlation. Thus, the media richness partially influent citizen engagement.

Hypothesis 4 mainly asked that whether the longer posts of Wuhan’s local government information release would result in a higher engagement rate. As the model result showed, text length was significantly positively associated with citizen engagement, especially with comments and likes, which means people tend to discuss the long text post.

Hypothesis 5 assumed that people tend to engage with original posts than non-original. The exam result is that posts containing sources negatively affected the three measures of citizen participation. It could be explained that people prefer to get involved in the original information.

## 5. Conclusions

### 5.1. Summary of Findings

This paper is the first to investigate how Wuhan’s local government used official social media for citizen engagement during the COVID-19 crisis. We used crawler technology to collect all the Weibo posts released by Wuhan Release from 31 December 2019, to 19 April 2020. We came to the following conclusion.

First of all, in terms of content types released in Wuhan, we have identified eight main types. Among them, thanks to workers and news reports, are the two most important information types that Wuhan release would like to post. During the outbreak, medical workers from all over China came to Wuhan, helping to cure and eliminate the epidemic. The government was committed to adopting a positive attitude and making these selfless actions public. News reporting is a routine job of a new media account. This will be followed by government measures, scientific guidance, and the release of epidemic data, which will be made transparent by making daily updates available.

Secondly, in response to Hypothesis 1, we used the method of median statistics to explore the trend of the content of different content types in different stages of the epidemic and found some special phenomena. From the result, we find that various types of messages play a different important role in every stage of the crisis. However, the information on refuting a rumor was always essential for the crisis response. This suggests the authorities should release information depending on the varying needs of the public and the actual situation of the crisis. Stopping the spread would be a key step throughout the whole period of the crisis.

Thirdly, we use multivariate statistics to verify which influencing factors of Weibo can generate greater citizen participation (repost, reply, likes). Concerning Hypothesis 2, we find that the dialogic loop is partially associated with citizen participation. Because @other accounts provide users with a distinct form of dialogue, it positively influent repost and thump-up behavior of users. Because the analysis of the data shows that the form of forwarding is accompanied by short characters, sometimes even in the form of “forwarding Weibo”, indirect information will lead to the decline of citizen participation. The hashtag did not affect citizen engagement because 95.6% (*n* = 3412) of all posts contained one or more hashtags.

As for Hypothesis 3, the media richness of the Wuhan release is positively correlated with citizen participation. Among them, the addition of pictures and links to information has a significant positive effect, which is consistent with previous studies [1,59,79]. Photos and links convey information in a fast, intuitive way that produces more interaction (likes or reposts) than with other types of media. However, more videos will lead to fewer citizen commentary, combing with the concise and rapid characteristics of social media, people will not be to understand a specific case to open a video.

The findings show that for Hypothesis 4, the scale of content is significantly positively correlated with citizen participation. This is an exploratory variable, in previous studies, many people considered multiple variables, but in terms of length, there was little research on the impact of exploration content on citizen participation. We found that during the COVID-19 management process, the more words published by the government, the higher the users get engaged. This research result provides some references for future research.

Finally, as for Hypothesis 5, this research anecdotally pointed to a preference of the citizens for the Wuhan local government original information during the COVID-19. It could be explained that at first the COVID-19 crisis was a local health crisis, and the public prefers the information from local rather than other sources, because the former was more closely related with them.

### 5.2. Practical Implications

This study brings some evidence of the factors influencing citizen engagement on Weibo. Firstly, government departments should comprehensively take the crisis lifecycle into account when conducting crisis response, which including a clear understanding of the crisis situation and a full analysis of the public’s information demand on different stages of a crisis. Secondly, government agencies should selectively employ media richness theory and actively use the dialogic loop to enhance public engagement. Government accounts can take full advantage of social media functions, especially mentions @ and hashtags, the number of original posts, add pictures and text length appropriately, to increase interactions with the public, to improve the level of engagement. Therefore, the results can be used to optimize the government information release model during the COVID-19 crisis to explain the influence of antecedence on social media users. We also provide a new method to capture and analyze all Weibo data officially released by Wuhan local government during the crisis.

Additionally, our findings may have practical implications for community managers in the public sector who are responsible for online communication during crisis outbreaks. Thus, our research can help local governments improve their online communication strategies, revealing factors that lead to higher citizen responsiveness. It is also possible to improve the quality of the information release services provided by maintaining a smooth dialogue with citizens by meeting their needs.

### 5.3. Limitations and Future Research

This study has its limitations as well as offering some directions for future research. First of all, we exclusively focused on Weibo during a situation of COVID-19 in Wuhan. At the beginning stage of the crisis, the government may choose some communication strategies such as denial, evasion, and diminishment to maintain the image and reputation, which may be deleted as the main strategies changed, this phenomenon makes it difficult to have a complete data. Moreover, because of the heavy censorship, negative or sensitive comments may be screened or deleted, which decreased the engagement motivation of the citizens. However, the anonymous and networked nature of social media makes it impossible to have an overall control [102], and the citizens also came up with corresponding strategies under the limitations on their freedom. The government cannot limit the expressive power of the people [103]. In this research, we still could evaluate the engagement level through the citizen behavior, such as like, comment and repost. Secondly, another limitation that needs to be recognized is the influence factors with government information release and reposts, replies, and likes, all of which are considered proxies for citizen engagement, but combined with the actual context, they may have a different use. Such as the time of forwarding, the emotion of the reply [27], special expression, or symbols [104]. Further analysis is needed in future studies to reduce this bias. Thirdly, although this study was conducted at the local government level in Wuhan, its main contribution can be extrapolated to other regions and applied to comparisons with other countries and international regions, which will improve the generality and understanding of the results. Findings from this study can represent a starting point for further understanding these phenomena in a more systematic fashion.

## Figures and Tables

**Table 1:** Content types.

Content Type	Post Content
News report	Answer the reporter’s question. The daily news. Live text.
Notice release	Activity cancellation. Charity publicity. Medical resources use publicity
Government measures	Measures taken by the central, provincial, municipal, district, county governments
Epidemic data release	Cases. Hospital beds. Cured number. Medical watch. Death
Scientific guidance	Scientific knowledge. Expert analysis. Protective measures
Thank the workers	Medical, military, police and all fields workers. Case presentation
Dispel rumors	Dispel rumors. Tell the truth
Encouragement	Encouragement. Look ahead. Positive energy.

**Table 2:** The divide of 22 groups of date.

1	2	3	4	5	…	18	19	20	21	22
12.31–1.4	1.5–1.9	1.10–1.14	1.15–1.19	1.20–1.24	…	3.25–3.29	3.30–4.3	4.4–4.8	4.9–4.13	4.14–4.19

**Table 3:** Percentage of content types.

Content Type	Percentage
Thank the workers	22.89%
News report	22.11%
Government measures	14.91%
Scientific guidance	12.40%
Epidemic data release	11.01%
Notice release	7.73%
Encouragement	6.15%
Dispel rumors	2.81%

**Table 4:** Correlations.

Factors	Repost		Commitment		Likes	
	Correlation Coefficient	Sig. (2-Tailed)	Correlation Coefficient	Sig. (2-Tailed)	Correlation Coefficient	Sig. (2-Tailed)
“@”Mentioned	−0.045 **	0.006	0.001	0.974	0.049 **	0.003
Hashtag	−0.038	0.245	0.021	0.515	0.011	0.727
Picture	0.002	0.882	0.034 *	0.041	0.020	0.221
Video	−0.032	0.051	−0.110 **	0.000	−0.028	0.091
Web-links	−0.070 **	0.000	0.120 **	0.000	0.051 **	0.002
Text length	−0.008	0.630	0.101 **	0.000	0.087 **	0.000
Information source	−0.079 **	0.000	−0.090 **	0.000	−0.065 **	0.000

**. Correlation is significant at the 0.01 level (2-tailed). *. Correlation is significant at the 0.05 level (2-tailed).

## Data Availability

The data presented in this study are available on request from the corresponding author.
